# Management of Temporomandibular Joint Ankylosis Using Temporoparietal Fascia Flap

**DOI:** 10.29252/wjps.10.2.93

**Published:** 2021-05

**Authors:** Mahdi Gholami, Ali Labafchi

**Affiliations:** 1 Associate Professor, Department of Oral and maxillofacial surgery, Faculty of Dentistry, Mashhad University of Medical Sciences, Mashhad, Iran.; 2 Dental student at MUMS, Student Research Committee, Faculty of Denti-stry, Mashhad University of Medical Sciences, Mashhad, Iran.

**Keywords:** Temporoparietal Fascia Flap, Temporomandibular Joint, Ankylosis

## Abstract

The adhesion of mandibular condyle to the glenoid fossa by bone or fibroblastic tissue is called temporomandibular joint (TMJ) ankylosis. Trauma and infection are the main reasons for affecting TMJ ankylosis. TMJ arthroplasty is the treatment of choice for this situation. This study was aimed at reporting a new technique for the treatment of chronic TMJ ankylosis caused as a result of a car accident in patients who referred to Ghaem Teaching Hospital, Mashhad, Iran. The primary Maximum Mouth Opening (MMO) of the patient was equal to 2.5 mm. Interpositional arthroplasty was done using Temporoparietal Fascia Flap (TPFF) based on the superficial temporal artery. The MMO was increased to 35 mm after regular post-operative physiotherapy and a one-year follow-up. There was no sign of reanykylosis during this time. Interpositional arthroplasty using TPFF could be an appropriate treatment option. TPFF is thin and has a reliable blood supply. It seems that TPFF has less complication compared to other Interpositional materials like temporalis muscle flap.

## INTRODUCTION

Temporomandibular joint (TMJ) ankylosis is a condition caused by adhesion of mandibular condyle to the glenoid fossa by bone or fibroblastic tissue. This situation can cause a lot of disturbances as well as difficulties in mastication and speech problems. Also, this affects oral hygiene and normal growth of facial bone in growing subjects. It is worth mentioning that the consequences of this problem in children are more catastrophic compared to adults. Trauma, infection, systemic diseases and previous TMJ surgery are potential causes of this disease. Among those, trauma is the most common cause and infection is the second. According to the previous studies (1-3). TMJ ankylosis can be diagnosed by plain films, orthopantomograms (OPG), Computed Tomography (CT) scans and MRI (4). 

The position of TMJ, the involvement of tissues, the extent of adhesion and rigidity of bones are the characteristics that are used for the classification of TMJ ankylosis to four types (4). 

Surgery is the treatment of choice for TMJ ankylosis. Gap arthroplasty and interpositional arthroplasty are two common and effective techniques. Gap arthroplasty is the oldest method of treatment. Although, this method is less time consuming and more economical, the risk of reanykylosis is higher after this operation (5). In order to avoid reanykylosis, it has been suggested to use interpositional materials such as temporalis fascia or muscle, fascia lata, skin, ear cartilage, fat and temporoparietal fascial flap (TPFF) (6). It has been shown that Interpositional arthroplasty has better results in treating TMJ ankylosis (5). TPFF is thin, flexible and well vascularized tissue that has been used for treatment of verity disorders in the head and neck regions (7). In 1898, Brown W used TPFF for the rehabilitation of horse’s external ear. Also, G.H. Monks used it for the reconstruction of the lower eyelid (8). 

Auteurs used various names such as temporoparietal fascia, superficial temporal fascia, epicranial aponeurosis, and galeal extension to describe this fascia. TPF is just below the hair follicles and subcutaneous fat. It is more superficial than the temporalis fascia and muscle. Loose areolar tissue has existed between these two fascias. Also, this layer is contained of Superficial Muscular Aponeurotic System (SMAS) and superficial temporal artery and vein. Due to the proximity of these layers, precise dissection is required (9, 10). 

The aim of this study is to report a successful method for the treatment of TMJ ankylosis done by Interpositional arthroplasty using TPFF and introducing the benefits of this method.

## REPORT OF CASE

A 54 –year- old man with general health who had an accident 13 years ago referred with the chief complaint of minimal mouth opening and the inability of chewing. The patient’s ethical consent form was signed and approved by the patient. 

**Figure 1:**
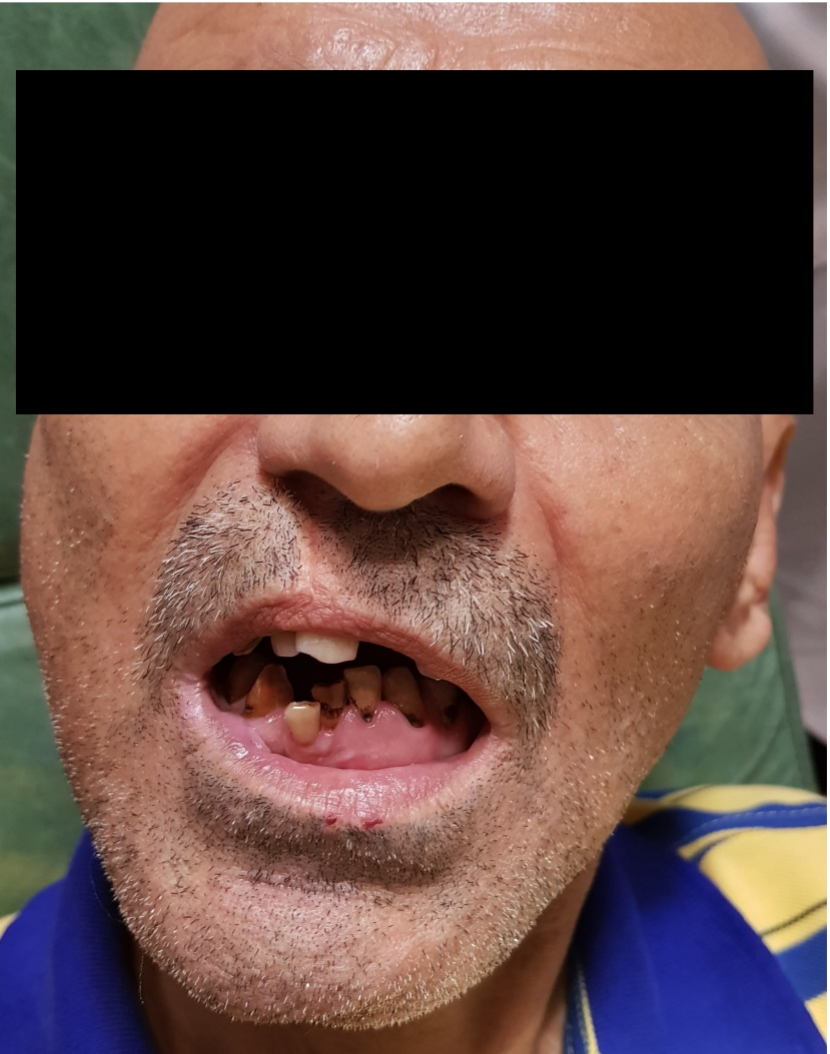
2.5mm MMO whit poor oral hygiene

The previous surgeon has done open reduction and internal fixation of symphysis fracture and close reduction of the right intra-capsular condylar fracture. Although the symphysis has been healed uneventfully, TMJ ankylosis appeared subsequently. Clinical examination revealed a Maximum Mouth Opening (MMO) of 2.5 mm with poor oral hygiene (Figure 1).

**Figure 2:**
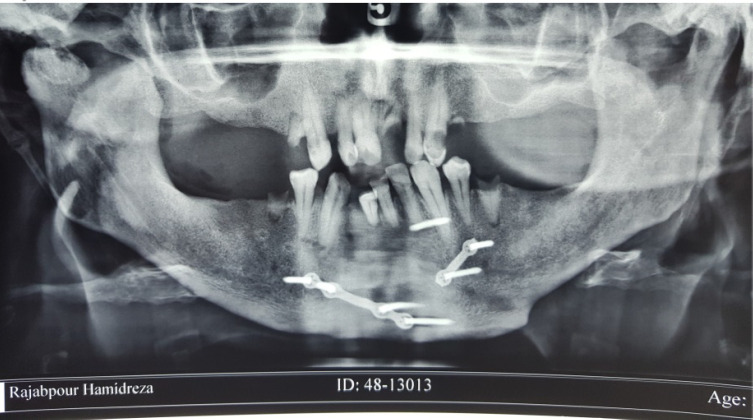
The radiographic view of right TMJ ankylosis

Radiographic investigations, including Orthopantomogram (OPG) and Cone Beam Computed Tomography (CBCT) revealed bony ankylosis of the right TMJ (Figure 2). 

Interpositional TMJ arthroplasty using TPFF was considered as the treatment plane for resolving the problem.

**Figure 3a, b:**
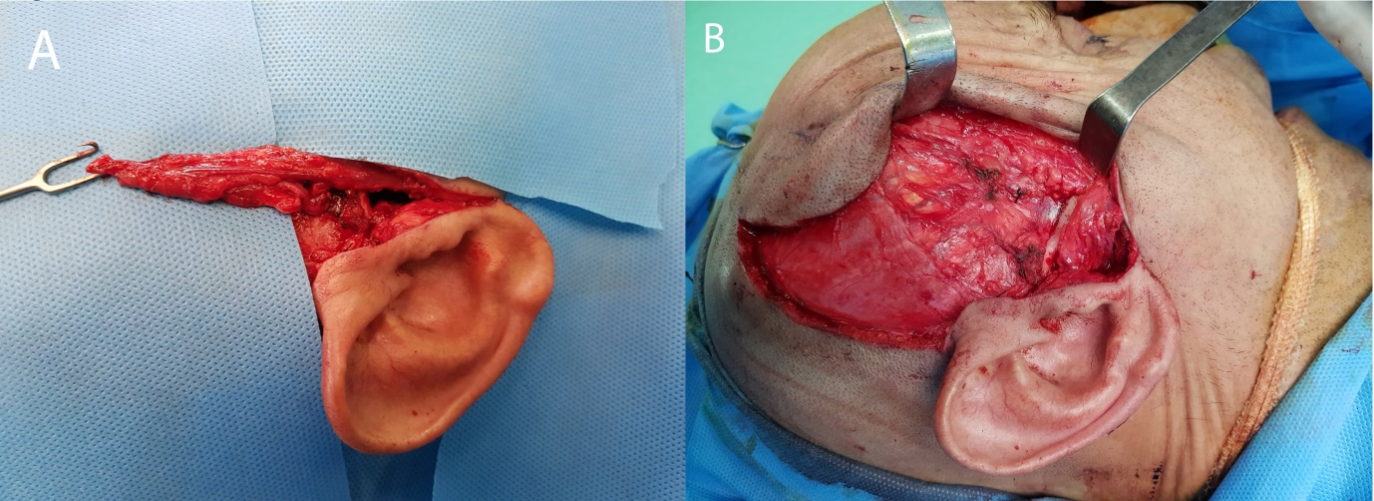
TPFF was elevated and rotated in to the created gap between newly formed condyle and temporal bone

Under general anesthesia and after shaving the right temporal region, the Bramley al-Khayat incision was made and through the sub follicular dissection, the superficial temporal artery and vein were exposed meticulously and the vascular pathway was followed within the temporoparietal fascia. By extending the incision to the preauricular area, the right condyle was exposed and the ankylosed condyle was released from the temporal bone using a fissure bur. We shaped a new condylar head using a number 4 round bur. After this stage, the MMO was increased up to 20 mm intraoperatively. In order to prevent reankylosis, Interpositional arthroplasty was done using TPFF based on the superficial temporal artery. The flap with 1×3 cm dimensions was elevated and rotated into the created gap between the newly formed condyle and temporal bone (Figure 3. A,B).

It was stitched and secured in place to the rest of the lateral pterygoid muscle and retrodiscal ligaments. After one week, the IMF was released and active physiotherapy was started using TENS and ultrasonic for ten sections. During the one year follow -up, there was no sign of reanykylosis, and the MMO of the patient has been increased up to 35 mm. 

## DISCUSSION

The temporoparietal facial flap is widely used in the head and neck region both as a free or pedicle flap. It is thin and has good blood supply Compared to various tissue layers such as temporalis muscle and fascia or parietal (9). 

Orbits, oral cavities, auricular, mandibular, and mastoid are the regions that we can use TPFF for reconstruction. TPF is a good choice for nasal reconstruction, and it can be used as a lining for cartilage coverage and nasal dorsum reconstruction (9). 

Reankylosis is a common sequel resulted from gap arthroplasty. Chossegros et al. found that the type of interposition material is an important predictor of reanykylosis (11). Interposition materials such as acrylic, auricular cartilage, costochondral and fat grafts, muscle or myofascial flaps are widely used for this purpose. Some of the disadvantages of alloplastic materials are displacement and Infection. Although, the immune response to the foreign body increases the risk of transplant rejection. The costochondral grafting technique is used widely as interposition material as a result of functional adjustment and growth potential. Despite the fact that unpredictable growth may cause unacceptable results. Fat grafting is another material that is a nonvascular tissue and does not survive for a long time (8). 

Temporalis fascia is another interposition material that was reported by Smith et al. in 1872 for the first time. It has a reliable blood supply, the risk of damage to the facial nerve branches is low during dissection of this flap and if a large amount of graft is needed, both fascia and muscles can be used (12). Studies showed conflicting results. Umeda et al. used axial temporalis fascia and muscle flap for 81 patients with TMJ ankylosis. 7 patients showed the symptoms of reanykylosis. They concluded that fascia and muscle should be dissected carefully (13). K. Su-Gwan in 2001 used. No sign of reanykylosis was observed (14). 

TPFF has several advantages including easy access and proximity to the TMJ area. It can be adjusted easily to fit the defect and has a reliable blood supply which leads to a decrease in the chance of necrosis and reanykylosis (8). 

Crawley WA et al. used TPFF and costochondral rib graft for reconstruction of TMJ ankylosis in 11 patients with different causes, including; tumor, trauma, and failed prosthetic joint replacement. During a seven-year follow-up, they showed the reconstruction’s stability without any Joint pain (15). 

Mokal et al. used 82 cases of TPFF in 71 patients. 29 people, including 25 children and 4 adults among TMJ ankylosis patients, were treated by interpositional arthroplasty. The interposition material for pediatric patients is a costal cartilage graft covered by pedicled TPFF and the material includes folded TPFF without and costal cartilage graft for adults. All patients had appropriate mouth opening and they had no sign of recurrence after 2 years follow-up (7).

## CONCLUSION

In conclusion, TPFF is a thin and flexible flap with good blood supply. It also has a suitable position in order to be used in surrounding defects. TPFF has wide usage in various clinical conditions such as orbits, oral cavities and auricular reconstruction. It can be used in Interpositional TMJ arthroplasty to treat TMJ ankylosis. According to this case, TPFF is a reliable interposition material for treating TMJ ankylosis. Randomized controlled clinical trials are needed to compare TPFF with Temporalis muscle/fascia for the treatment of TMJ ankylosis.

## ETHICAL APPROVAL

All procedures performed in this study involving the human participant were in accordance with the ethical standards of our institutional research committee and with the 1964 Helsinki declaration. The patient’s ethical consent form was signed and approved by the patient. All procedures used in this research were approved by the Ethical Committee of Mashhad University of Medical Sciences.

## FUNDING

None. This study was self-funded.

## CONFLICT OF INTEREST

The authors have no conflict of interests to declare.

## References

[1] Rajurkar SG, Makwana R, Ranadive P, Deshpande MD, Nikunj A, Jadhav D (2017). Use of Temporalis Fascia Flap in the Treatment of Temporomandibular Joint Ankylosis: A Clinical Audit of 5 Years. Contemporary clinical dentistry..

[2] Goswami D, Singh S, Bhutia O, Baidya D, Sawhney C (2016). Management of Young Patients with Temporomandibular Joint Ankylosis-a Surgical and Anesthetic Challenge. The Indian journal of surgery..

[3] Katsnelson A, Markiewicz MR, Keith DA, Dodson TB (2012). Operative management of temporomandibular joint ankylosis: a systematic review and meta-analysis. Journal of oral and maxillofacial surgery : official journal of the American Association of Oral and Maxillofacial Surgeons..

[4] Movahed R, Mercuri LG (2015). Management of Temporomandibular Joint Ankylosis. Oral Maxillofac Surg Clin North Am..

[5] Ma J, Liang L, Jiang H, Gu B (2015). Gap Arthroplasty versus Interpositional Arthroplasty for Temporomandibular Joint Ankylosis: A Meta-Analysis. PLoS ONE..

[6] Tellioglu AT, Tekdemir I, Erdemli EA, Tuccar E, Ulusoy G (2000). Temporoparietal fascia: an anatomic and histologic reinvestigation with new potential clinical applications. Plastic and reconstructive surgery..

[7] Mokal NJ, Ghalme AN, Kothari DS, Desai M (2013). The use of the temporoparietal fascia flap in various clinical scenarios: A review of 71 cases. Indian Journal of Plastic Surgery : Official Publication of the Association of Plastic Surgeons of India..

[8] Jaquet Y, Higgins KM, Enepekides DJ (2011). The temporoparietal fascia flap: a versatile tool in head and neck reconstruction. Current opinion in otolaryngology & head and neck surgery..

[9] Demirdover C, Sahin B, Vayvada H, Oztan HY (2011). The Versatile Use of Temporoparietal Fascial Flap. Int J Med Sci..

[10] Qiao J, Yu B, Gui L, Fu X, Yen CK, Niu F (2018). Interpositional Arthroplasty by Temporalis Fascia Flap and Galea Aponeurotica Combined With Distraction Osteogenesis: a Modified Method in Treatment of Adult Patients With Temporomandibular Joint Ankylosis and Mandibular Dysplasia. The Journal of craniofacial surgery..

[11] Chossegros C, Guyot L, Cheynet F, Blanc JL, Cannoni P (1999). Full-thickness skin graft interposition after temporomandibular joint ankylosis surgery A study of 31 cases. International journal of oral and maxillofacial surgery..

[12] Smith JA, Sandler NA, Ozaki WH, Braun TW (1999). Subjective and objective assessment of the temporalis myofascial flap in previously operated temporomandibular joints. Journal of oral and maxillofacial surgery : official journal of the American Association of Oral and Maxillofacial Surgeons..

[13] Umeda H, Kaban LB, Pogrel MA, Stern M (1993). Long-term viability of the temporalis muscle/fascia flap used for temporomandibular joint reconstruction. Journal of oral and maxillofacial surgery : official journal of the American Association of Oral and Maxillofacial Surgeons..

[14] Su-Gwan K (2001). Treatment of temporomandibular joint ankylosis with temporalis muscle and fascia flap. International journal of oral and maxillofacial surgery..

[15] Crawley WA, Serletti JM, Manson PN (1993). Autogenous reconstruction of the temporomandibular joint. J Craniofac Surg..

